# Self-Healing Polymeric Composite Material Design, Failure Analysis and Future Outlook: A Review

**DOI:** 10.3390/polym9100535

**Published:** 2017-10-20

**Authors:** Keletso Mphahlele, Suprakas Sinha Ray, Andrei Kolesnikov

**Affiliations:** 1 DST-CSIR National Centre for Nanostructured Materials, Council for Scientific and Industrial Research, Pretoria 0001, South Africa; kmphahlele@csir.co.za; 2 Department of Chemical, Metallurgical and Material Engineering, Tshwane University of Technology, Pretoria 0001, South Africa; kolesnikova@tut.ac.za; 3 Department of Applied Chemistry, University of Johannesburg, Doornfontein, Johannesburg 2028, South Africa

**Keywords:** polymer composites, self-healing, cracks, modelling, review

## Abstract

The formation of micro-cracks and crack propagation is still an acute problem in polymer and polymer composites. These micro-cracks usually occur while the materials are manufactured or serviced. The development and coalescence of these cracks reduces the lifespan and brings about a catastrophic failure of the materials. Novel scientific research on polymeric self-healing is emphasised in a number of publications, which consist of contributions from many of the prominent researchers in this area. Progress in this field can eventually enable scientist to construct new flexible materials that both monitor the material’s integrity and repair the deformed material prior to the occurrence of any fatal failures. This report describes recent trends that have been used in material science and computational methods to mitigate the development of micro-cracks and crack propagation in polymer composites.

## 1. Introduction

**Figure 1:**
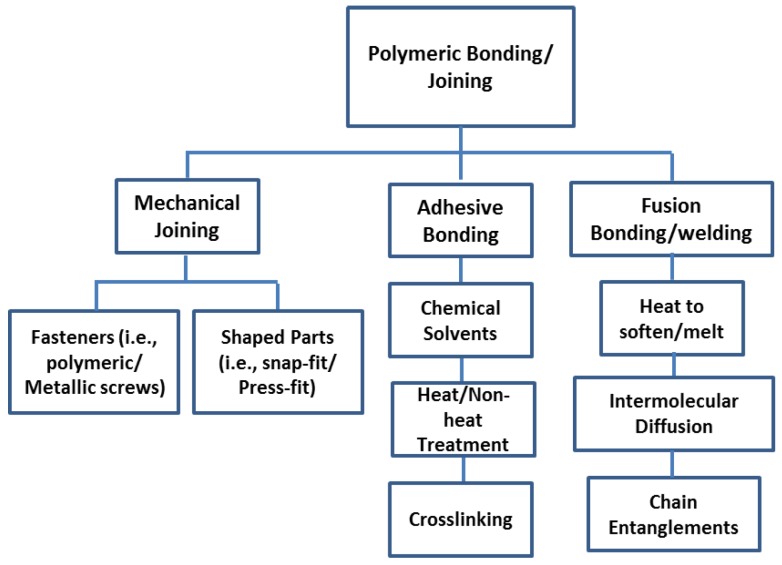
Possible joining techniques for polymeric materials. The concept has been redesign on the basis of ref. [6].

Composite materials are a judicious combination of two or more materials with diverse properties. These materials work together to give the composite distinctive properties [1]. The assemblage of sub-components is critical when manufacturing polymer composites (see Figure 1). A model structure would be engineered without hinges/joints because hinges could weaken and add extra weight to the polymeric composites [2]. Accepted assembling technologies for metallic structures are indirectly applicable to composites material [2,3]. Mechanical joining uses separate fasteners [4], such as metallic or polymeric screws, or it depends on the combined design elements that are shaped into parts, such as snap-fit or press-fit joint [5,6]. Adhesive bonding involves an adhesive placed between an adherent, which serves as the material that combines the parts and transmits the load through the joint [6,7,8,9,10]. Chemicals that are utilised to the surface used in adhesive bonding are difficult to regulate in engineering structures; these chemicals directly affect the strength and stability of bonded joints [2,11,12,13]. Sensitivity to storage is another aspect of surface treatment, and current recycling requirements induce industries to select adhesive structures that are biodegradable [2]. In fusion bonding/welding, heat is employed to soften or melt the polymer at the interface to allow polymer intermolecular diffusion across the interface and chain entanglements [14] to impart strength to the joint [6]. By choosing a suitable combination of matrix and reinforcement material, a new material can be made that precisely meets the requirements of a specific application [15]. In this study, we are interested in fibre-reinforced composites.

The study of fibre-reinforced composite materials has matured rapidly since their introduction in the 1970s [15,16]. Approximately 20 million tons are manufactured annually for a range of products in aerospace and other industries [16,17,18,19]. Carbon fibre composite materials are strong and lightweight [20], however, these materials are prone to delamination, micro-cracking and fibre debonding deep within the structure. Therefore, there are concerns relating to the structural reliability of composite materials after impact loading. Exposure to harsh environment can cause the degradation of polymeric components, and small-size defects such as micro-cracking may be generated during manufacturing or servicing of a polymeric component, which cause disastrous failures of the materials, considerably reducing the lifespan of the structures [20,21]. These cracks are particularly challenging to identify and restore by traditional methods [16,21].

Conventional techniques such as non-destructive evaluation (NDE), non-destructive testing (NDT) and non-destructive inspection (NDI) [22,23,24] are used to detect local damage and identify emerging failures in critical structures [25] (i.e., ultrasonic infrared thermography, electromagnetic testing, X-ray tomography, computerized vibro thermography etc.) [26]. These techniques have been commercialised and approved by engineers for replacing visible damages on polymeric materials, and if the deformation is too serious, the structural constituent is changed completely [27,28]. If localized delamination occurs, it may be restored by injecting epoxy resin through an entry hole into the damaged part. Another conventional repair technique is to employ a strengthening bolt or patch fused to the composite structure [29]. These methods are momentary resolutions to extend the lifespan of the structure, and they require manual intervention and observations of the damage to for repair [30]. Therefore, these conventional restoration approaches are time-consuming, costly and ineffective for healing the micro-cracks embedded into the structure during its service lifecycle [31]. The ultimate goals for engineers/scientist is to build everlasting composites with an integral ability to self-diagnose, self-control and self-heal where issues like operation safety and lifecycle cost can be avoided [32].

The proposed theory of self-healing is that the deformed structure is repaired by materials already confined within it [16], self-healing materials are motivated by biological systems, which are evolutionarily optimized functional systems [33]. This concept of autonomic healing in biological systems has inspired continuous efforts to mimic biological materials and to integrate self-healing abilities into polymeric composites for engineering applications [34].

## 2. Self-Healing Polymeric Materials and Traditional Repair Methods

While there has been great advancement in self-healing polymeric materials, we shall first discuss some of the traditional methods used to repair damage in polymeric methods. 

### 2.1. Bonded Composite Repair

**Figure 2:**
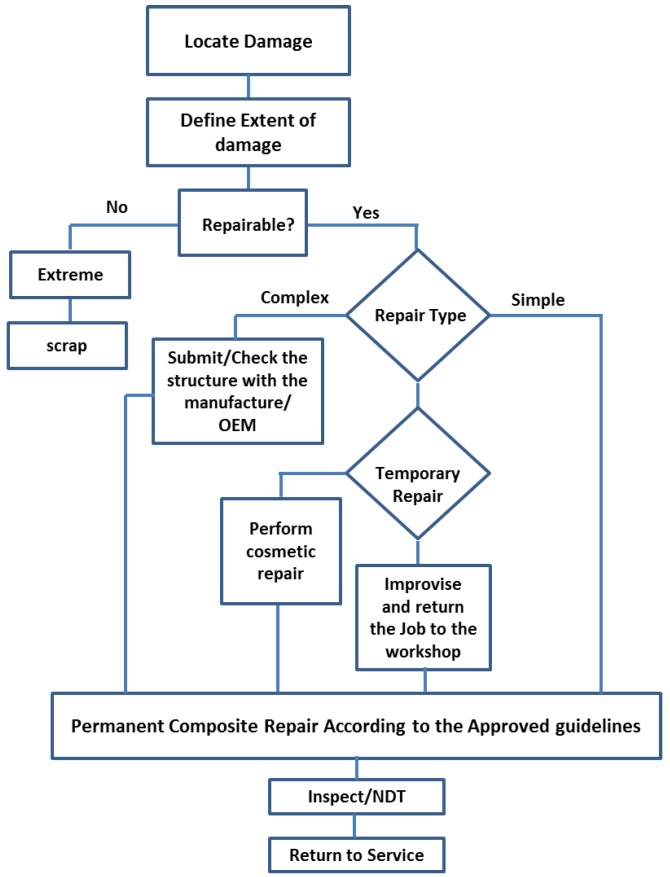
Key stages of composite repair. The concept has been redesign on the basis of ref. [46].

In the 1970s, the Aeronautical and Maritime Research Laboratory (AMRL) in Australia established the composite repair technology [35], Figure 2 shows the key stages of composite repair. A lot of research has focused on prolonging the structural life of deformed structures; extending the life of old aircraft structures is emphasised using numerous remedial methods [36]. The remedial approach depends on the degree of damage and static strength and thickness, stability, toughness requirements, weight and balance, and aerodynamic smoothness. For aerospace maintenance, operational temperature and environment are some of the key criteria that determine the type of structural repairing methods [35]. Structural adhesive bonding repair technology is a composite repair method in which the repair materials must overlay, and be effectively bonded to the plies of the original laminates [37]. Secondary patching with high performance structural adhesive is employed in the assembly of composite material [37]. Three approaches are available for restoration of bonded material composite: single or binary-sided doubler patches [38,39], tapper sanded or scarf repair, and stepped sanded repair [40,41].

**Figure 3:**
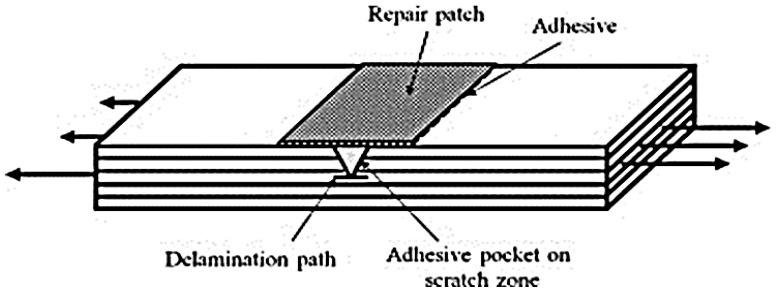
Schematic of a scratched specimen with overlay repair. Reproduced with permission from ref. [45].

Patches have been accepted as a quick repair method for aircraft skins, boat hulls and internal tube of tyres. The composite patch repair method is temporary and is meant to extend the operational life of the structures [35,42]. The thickness of the original laminates is fabricated with filler plies and the repair materials are bonded to the surface of the laminates. Proper design of the repairs requires that the patch absorbs a considerable fraction of the load forced in the locality of the crack and that the patch does not debond from the structure during service [43]. The success of the repair technique depends on the coherence between the materials of the base structures, and patch, adhesive used, surface treatment and finally, skills of the repair operatives [36,44]. A graphic representation of the specimen with the repair patch is shown in Figure 3. The disadvantages of the composite reinforcement repairs is the sustainability and toughness of the patch constituents, patch loss detectability, the inhibition of crack growth at the crack tip, hydro-thermal consequences, and the optimal patch form [45].

**Figure 4:**
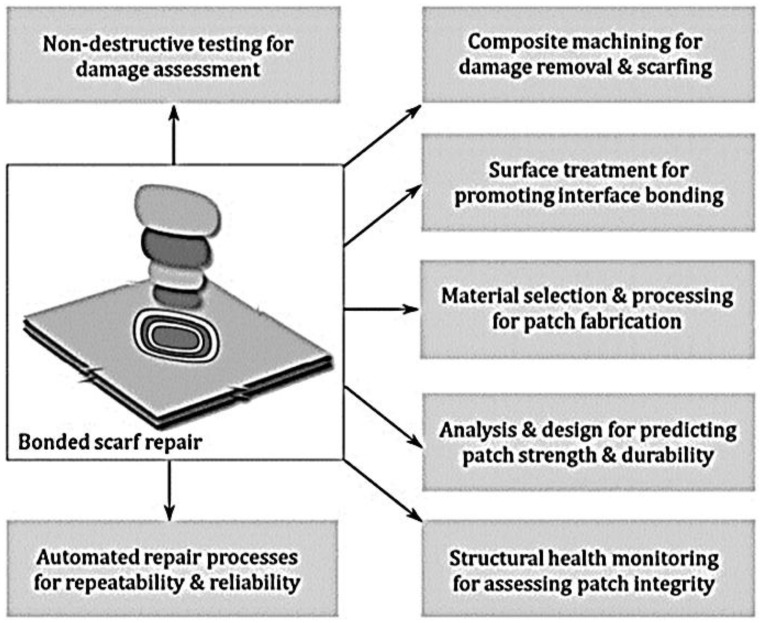
Research challenges for developing robust bonded scarf remedial technology. Reproduced with permission from ref. [37].

Taper sanded or scarf repairs or step scarf repair (i.e., Figure 4) are the favoured techniques of remediation to repair the load carrying ability of the deformed composite structures to the original strength [40,45], and scarf maintenances is fairly common for reducing aerodynamic disruption [7,45]. Scarf repair offers both structural strength and a flush surface [41,47], therefore, it is often used for aircraft composite repair [35,39]. In designing a composite restoration, characteristics such as robustness and operative conditions should be considered to ensure the efficiency and structural reliability of the repair [37].

**Figure 5:**
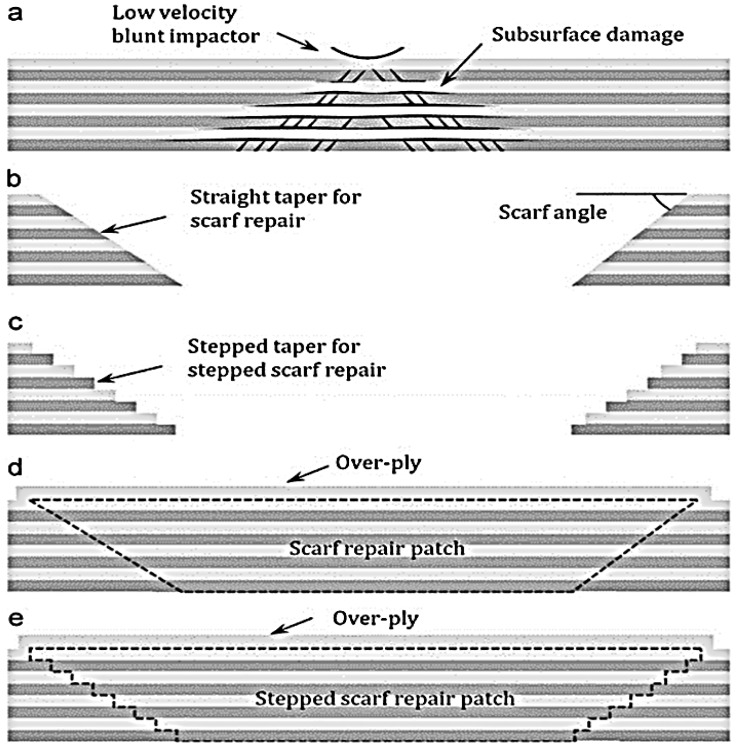
Bonded composite restoration: (**a**) subsurface deformation usually prompted by low velocity dull impactor; (**b**,**c**) composite machining to achieve level and stepped taper for scarf and stepped scarf restoration; (**d**,**e**) scarf and stepped scarf remedial patches. Reproduced with permission from ref. [37].

Katnam et al. discussed a diagram that showed fundamentals of structural bonded scarf repair, which comprises of six classes: (a) deformation assessment; (b) structural removal; (c) surface preparation; (d) patch fabrication; (e) design; and (g) observation and automation, shown in Figure 5 [36]. Structural damage requires accurate evaluation to perform an adequately bonded repair [47]. Structural bonded repairs offer improved stress transfer mechanisms, joint effectiveness and aerodynamics [35,37]. In order to have a comprehensible and cost effective aircraft composite remedial strategy, it’s important to design robust, consistent and repeatable bond remedial technologies [36]. There is a necessity for researcher the improve the existing composite remedial technologies in areas such as advanced NDT for deformation evaluation [48,49,50], unconventional composite machining for structural removal [51,52], advanced surface treatments for boundary bonding, controlled cure conditions for reinforcement manufacture, precise investigation and design of improved repairs [53], condition observation and automation for dependable and repeatable restoration [37]. However, currently, determining damage mechanisms in composite materials are complex because the damage usually manifests itself internally [34,54], and NDI faces challenges in the correct and reliable evaluation of the deformation [10,37,54].

### 2.2. Welding/Fusion Bonding

Welding is a sculptural procedure that enables the re-joining of cracked structures or blending new constituents to the deformed area or the polymeric composite, by initiating coalescence [31,55]. This is usually done by melting the work fragments and adjoining a filler material to form a puddle of liquefied material, and when it cools down it becomes a robust joint. Pressure is occasionally employed in along with heat to generate the weld. In the structural engineering division, there are about 730,000 permanent and 5.5 million welding related occupations in Europe [55]. There are new kinds of welding processes and goods, some of these methods used are shielded metal arc welding (SMAW) [56], gas tungsten arc welding (GTAW) [57], flux-cored arc welding (FCAW) [58], submerged arc welding (SAW) [59], and electro slag welding (ESW) [55].

**Figure 6:**
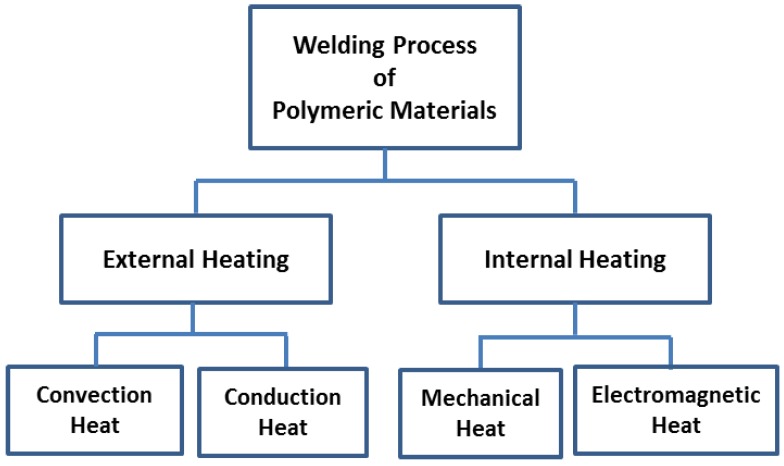
Classification of welding techniques for polymeric materials. The concept has been redesign on the basis of ref. [6].

Thermoplastic matrix composites (TPCs) have benefits compared to thermosetting-based composites (TSCs), which includes an improved fracture toughness, lower moisture absorption, potential for condensed lifecycle cost, and biodegradability [6,9]. This is because, with TSCs, a chemical reaction occurs during processing, and curing; the resultant is an irreversible cross-linking reaction in the mould [60]. Thus, heating cannot reshape moulded TSCs, because degradation occurs [61]. Welding processes in TPCs and composite materials are divided into two categories: internal and external heating (i.e., Figure 6). Internal heating is categorized into mechanical and electromagnetic heating [6]. External heating methods rely on convection and conduction to heat the weld surface (i.e., heating tools such as hot plates, hot gases, extrusion, implant induction and implant resistance welding) [6,61].

Although this method offers great potential in repairing composites materials, most welding processes generate various fumes and gases [62]. Welding gases are metal containing aerosols consisting of particles created through complex vaporisation–condensation oxidation procedures during welding [63,64]. Health issues related to metal gases depends on the type of metals present in the fumes; therefore, there are concerns that these gases might cause diseases, such as metal fume fever, to long-term lung damage and neurological disorders, (i.e., lung cancer and Parkinson’s disease). Gas phase contaminants produced during welding procedures; may release carbon dioxide (CO_2_), carbon monoxide (CO), nitrogen oxides (NO_x_), sulfur dioxide (SO_2_) and ozone (O_3_) [55].

### 2.3. In-Situ Curing of New Resin

Curing of polymers is a complicated procedure, which has a strong influence on the type of applications it can be used in [65]. In the first stage, which occurs during mixing in a pot, the resin and hardener are brought together as a uniform liquid, initiating a chemical reaction. A liquefied polymer is gradually transformed into a viscoelastic solid which is formed by cross-linking of the initially short polymer chains. In the second phase, the gelation stage, the viscoelastic polymeric solid cross-linking density (resin and hardener molecules are interconnected together) is adequate for the liquid to achieve the consistency of a viscous gel until it reaches a state when it is no longer a liquid and has lost a its ability to flow. The last stage is when the surface is ‘active’ and can attain additional layers of resin and still form a decent bond. This method is comparable to patching, as a new material is employed to reinforce the mechanical strength [31,66]. Sometimes, patching methods include the addition of an uncured resin to an excavated section of the original polymeric material. The uncured resin disperses into the deformed constituent and the adhesive forces hold the patch in place [31,66,67].

While, traditional repair methods can effectively repair visible or external damage, these are time consuming, costly and need reliable detection techniques and experienced workers [32]. These techniques are not effective in repairing internal or invisible micro-cracks [68,69]. Thus, the development of self-healing materials proposes a new path towards benign, long long-lasting materials and will fill a technology gap in alleviating damage in composite materials.

## 3. Self-Healing Concept

Repairing is defined as a process of restoring something damaged, faulty or worn out to a good condition and healing is the process of restoration. Thus, self-healing means a mending of the original properties of the material after destructive actions of the exterior environment [70]. Synonyms such as self-healing and autonomic-healing are employed to describe such characteristics in the material [71]. The aim is to reduce the damage or restore the functionality and lifetime of the deformed part, system or device. Therefore, self-repair in principle is an automatically and autonomously initiated response to damage or failure [71,72]. Self-healing materials are motivated by living systems; biological systems/material are functional systems optimized by evolution [33]. This concept of autonomic healing in biological systems has inspired continuous efforts to mimic biological materials and to incorporate self-healing abilities into polymers and polymer nanocomposites for engineering applications [34,73].

Self-healing materials can be further subdivided into subclasses that are used to describe the self-healing properties; these classes are usually termed extrinsically and intrinsically self-repairing [33]. The extrinsically self-repairing process is based on exterior modules, such as micro-or nanocapsules with self-healing agents, which are deliberately enclosed into the matrix materials to create self-healing properties [33,66,74]. Whereas, intrinsic self-healing requires no healing agent, thus, the material itself possesses healing properties [66]. Intrinsically repairing materials are appealing to the science community as these materials are composed of dynamic bonding mechanisms, which theoretically are qualified for repetitively repair at the same deformed site [74].

**Figure 7:**
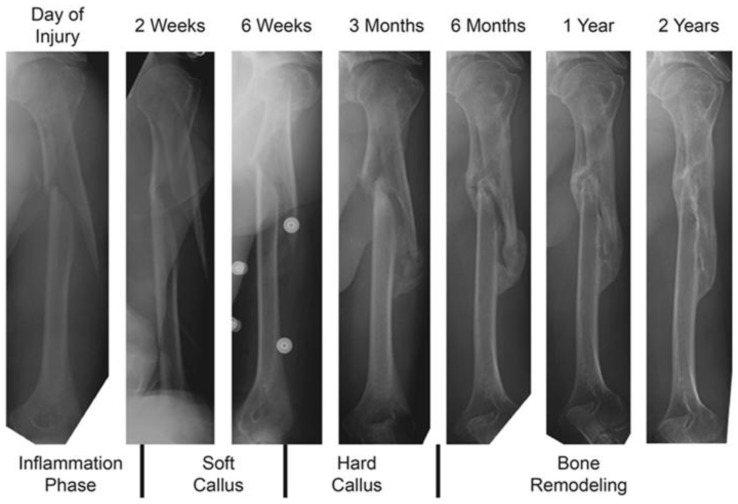
Representation of bone in bone healing. Reproduced with permission from ref. [80].

Polymeric self-healing is based on a three-step process. Polymeric self-healing can be considered analogous to the biological healing process in living creatures [75]. An example of this process is the process of self-repair of bone after injury, which is shown in Figure 7. The first stage is the formation of hematoma, the blood vessels, and tissues on the fragmented bone are torn, resulting in a hematoma mass of clotted cells between and around the fracture, developing and stabilizing the inflammatory cells to damaged sites. This step is followed by the formation of fibro-cartilaginous callus, which is characterized by the conversion of the hematoma to a fibro-cartilaginous mass to bridge the fracture. Next, bone callus formation occurs, where a fibrous bone (spongy bone) is formed. Finally, the woven bone of the hard callus matures and organizes into a trabecular structure to recreate the native pre-injury structures; this step is classified as the bone remodeling stage [72,76,77,78]. The process of repairing a fractured bone can be adapted in the polymeric self-healing process, where the first step is the triggering action that happens immediately after the occurrence of the damage/crack. The second step involves the transportation of the healing agent to an affected area, the third step is the chemical repair process, and finally is the remodelling of the healed polymer material [78,79].

**Figure 8:**
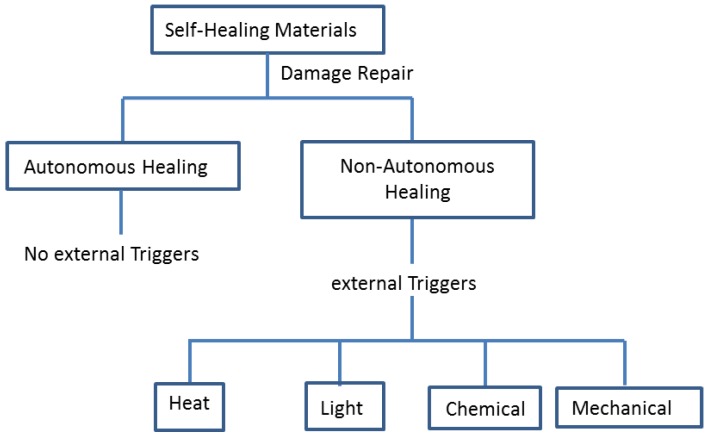
Illustrate the difference between autonomic and non-autonomic self-repairing process. The concept has been redesign on the basis of ref. [66].

There are several approaches used to synthesise self-repairing materials, depending on the material class. Self-repairing materials can be classified into two categories, depending on the necessary trigger and the nature of the self-repairing procedure (i.e., autonomic and non-autonomic) [33]. Autonomic self-repairing materials do not need an external stimuli, the damage itself is the stimulus for the repair. Examples include capsules or fibres with a healing agent which are deliberately enclosed in the matrix to create self-healing properties [66,74]. Whereas the non-autonomic self-repairing process requires external triggers such as light, heat, laser beam, chemical, mechanical, and so on, so healing can occur (Figure 8) [33,66]. 

## 4. Extrinsic and Intrinsic Self-Healing Materials

**Figure 9:**
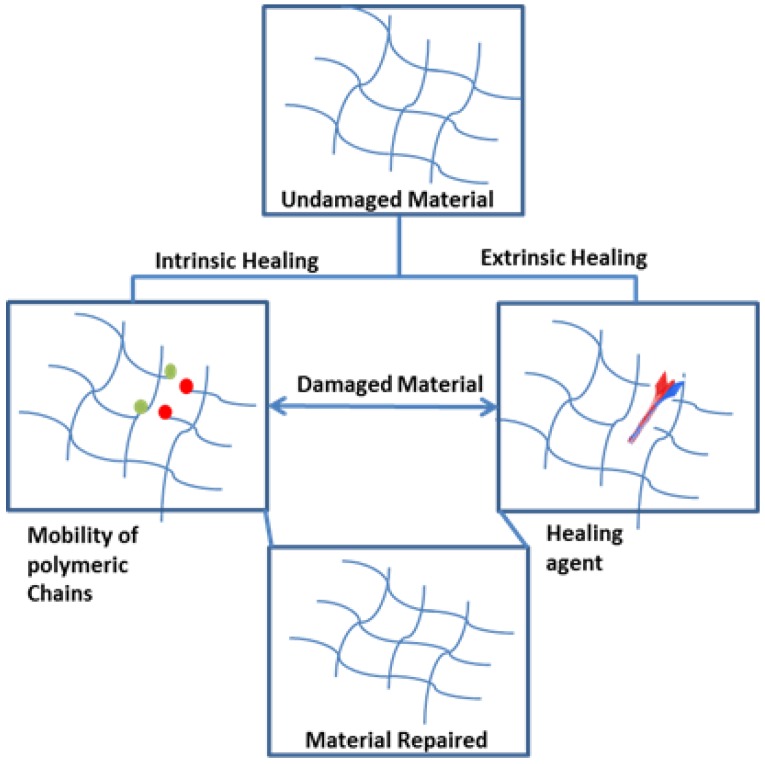
Distinction between extrinsic and intrinsic self-repairing materials. The concept has been redesign on the basis of ref. [81].

The difference between extrinsic and intrinsic materials is that in extrinsic healing the healing agents is used to facilitate the repair of a structure, and intrinsic healing is achieved by the material repairing itself by its own ability (see Figure 9).

### 4.1. Intrinsic Self-Repairing Materials

As discussed in the previous paragraphs, intrinsic self-healing polymers are capable of restoring molecular and macro-scale damages via a momentary local increase in the mobility of polymeric chains [33,68]. This behaviour is based on the precise molecular structures of the polymers that permit a marked step in (effective) inter-chain mobility upon the supply of modest amounts of energy such as temperature, static load, UV, and, finally, a method of repairing the chemical and physical bond strength upon removal of the stimulus follows. The advantage of intrinsic healing over extrinsic healing it that it relies on the prospect of full or partial repairing of the initial properties multiple times [68,82]. Intrinsic healing can be grouped into two wide classes: (a) dynamic covalent bonds [82,83]; and (b) supramolecular interactions, ref. [82] discussed below.

### 4.2. Intrinsic Self-Healing

This section discusses dynamics; which is defined as constitutional dynamic polymers, that is, polymeric entities whose monomeric constituents are connected through reversible networks and have therefore the ability to modify their composition by exchange and reshuffling of their components. They may be either of molecular or supramolecular systems depending on whether the connections are dynamic covalent bonds or non-covalent interactions [84]. 

The basic principle of dynamic covalent bond formation is that the bulk polymer comprises of covalent crosslinks [85,86] that experience distinct and complete reversible bond breaking and bond forming reactions during repair. Thus, the material inside the repaired region can be chemically indistinguishable from that of the body of the polymeric material [83]. Such a reversible modification in composition allows the structure to have great motorized attributes when they are in the polymer phase, and mobility when they are monomeric, oligomeric or non-crosslinked. These are the chief necessities for understanding of self-healing in materials because it permits mass transport of species towards the deformed area only when it is preferred [81]. Perhaps the most recognised chemical reaction used for intrinsic self-healing materials is the Diels-Alder (andretro-Diels-Alder) reaction [81,82]. The reaction relies on a trigger/stimulus like thermal energy to regulate of bond formation and breaking [87]. Three-dimensional cross-linked networks can be created using several systems based on the [4 + 2] Diels-Alder reaction and its reverse [87]. These structures comprise numerous dienophile and diene moieties of low molecular weight, dienes or dienophiles attached to polymer spines cross-linked with bi-functional low molecular weight cross-linker, or polymeric chains with both diene and dienophile. There are numerous chemical clusters that can react through the Diels-Alder mechanism, which include fulvenes, cyclopentadiene, anthracene and others, but the most explored involves furan as the diene and maleimide as the dienophile [88]. This is perhaps because of the rapid kinetics, which makes them attractive for prospective engineering applications [89]. Regarding the Diels–Alder chemical reactions, several di-enes and di-enophiles can be used but the furan–maleimide interaction with a repairing temperature range of 100–150 °C is best known [82,90]. Disulfide bonds are versatile and relatively easy to implement in existing networks. Disulfide encouraged healing has been established in conventional epoxy-based thermosets utilizing aliphatic disulfides with chains, with thiols as the precursors, and at mild healing temperatures (60–70 °C), in ambient-temperature elastomers based on aromatic disulfide metathesis and in hybrid sol–gel based coatings [91,92,93]. It is extraordinary to consider that although the reversible chemical principles of the Dies-Alder/reverse Diels-Alder reaction and disulfide bonds have been known for years, it has not been until relatively recently that these principles have been applied to yield self-healing polymeric materials [82].

The theory of supramolecular self-healing materials is dependent on the use of noncovalent, transient bonds to generate networks, which are capable to heal the damaged location, putting the aspect of reversibility and dynamics of a network and its fundamental supramolecular bonds in the spotlight for the understanding and design of self-healing polymers via this concept of self-healing. Furthermore, supramolecular interactions can affect material properties such as the polymers’ strength (moduli), its viscosity and flow, as well as the intrinsic organization of polymer chains [84,93,94,95,96]. Perhaps the two most studied interactions so far are those found in ionomers for ballistic [82,97] and coating applications and hydrogen bonding represented by the well-defined ureidopyrimidinone constituent and the use of randomly branched oligomers equipped with self-complementary and complementary hydrogen bonding groups [82].

The self-healing method of ionomers originates from the intrinsic chemical structure [94] and morphology of copolymers containing 15 mol % ions [98], and in which the bulk properties are directed by ionic interfaces within the polymer [99,100]. The subsequent reversible physical crosslinks joined with a compound microstructure have a great effect on the physical and mechanical properties of the polymer and reinforces the self-healing performance [99]. The formation of an order to disorder transition (Todt) efficiently produces a network polymer comprising of reversible crosslinks and is similar to a glass transition temperature in thermosets. The process can in principle repeat several times, thus, it is unique from other structures which typically cannot mend themselves continuously [98]. Zhang et al., reported using the thermal adhesiveness of poly(ethylene-co-methacrylic acid) (EMAA) copolymer, a new approach for a thermoplastic healing agent. EMAA particles (250–425 μm) are directly added into triethylene tetramine (TETA) cured diglycidylether of bisphenol A (DGEBA) epoxy resin. Impaired distinct edge tapered double and notched bars cantilever beams (TDCB) were repaired at 150 °C for 30 min to achieve up to an 85% recovery in critical stress intensity and above 100% recovery in maintainable peak load [16]. 

### 4.3. Extrinsic Self-Healing Materials

Capsule-based self-healing materials fall under the category of autonomic repairing materials. Thus, the healing agent is encapsulated until damage activates fissures and discharges the healant. There are several techniques used for encapsulation of reactive materials. For self-healing materials, interfacial, in situ and meltable dispersions are commonly used [69]. To date, self-healing materials involving embedded microcapsules have been widely investigated [97]. Materials such as micro-encapsulated dicyclopentadiene reacting with Grubb’s catalyst implanted in the polymer matrix to cure damage have been widely studied and reported. White et al. reported on microcapsules filled with remedial agent encapsulated in the polymer matrix. In this example, crack propagates to the microcapsules causing the restorative agent to stream into the crack. An embedded catalyst initiates polymerization of the restorative agent with up to 75% fracture toughness recovery reported [21]. The effects of size and concentration of microcapsules on the fracture toughness and healing effectiveness were described by Brown et al., they reported a 90% recovery of fracture toughness after healing [100]. Similarly, Kessler et al., utilised a comparable technique for to establishing up to 80% recovery in carbon fibre-reinforcement at high temperatures, with up to 45% recovery at ambient temperature [29]. Tao et al. described the use of a two-component healing agent which comprises an epoxy that was micro-encapsulated as a polymerisable remedial resin, ensuring miscibility between the healant and an epoxy based composites. The complex of CuBr_2_ and 2-methylimidazole (CuBr_2_(2-MeIm)_4_) was manufactured as the latent hardener of an epoxy restorative agent. The complex possesses lasting stability and dissociates into CuBr_2_ and 2-methylimidazole again at around 130–170 °C. A 111% recovery in fracture toughness of the epoxy, with no loss of fracture toughness paralleled that of neat epoxy [93]. 

Vascular self-healing materials are similar to the capsule-based method. The restorative agent is embedded in a network in vessels or hollow canals, which are interconnected until damage prompts self-healing. In this vascular self-healing materials, the interactions between the matrix materials, the healant, and the network materials play a vital role in the successful creation of a self-healing organization [67]. Vascular systems are arranged according to the connectivity of the vascular networks, leading to one, two and three-dimensional networks. Initial models of self-healing materials were reported by Dry et al., and White et al. In these studies, empty glass fibres packed with a healing agent were embedded in a polymer matrix. The healing agent kept in the hollow fibre is discharged into the deformed area to heal the composite upon rupture caused by mechanical loading of the hollow fibre. The chemical enclosed within the hollow fibres is typically adhesive or air-curing polymer/monomers. Repair using of this technique was verified in both impact and fibre pull-out testing method [97,101,102,103]. Bleay et al. studied a composite material self-repairing system. Hollow glass fibre composites were filled with an X-ray dense dye penetrant and one and two-part curing resin systems. These were then evaluated for the capability of both self-repair and improving damage recognition. A vacuum aided capillary action loading method was established and used to effectively pack the hollow fibres. Different treatments were applied to drive the resin out of the fibres after impact. Synchronising heat and vacuum proved to be the most effective technique. The damage recognition method was enhanced by using an X-ray opaque dye. It exposed both the damaged area and the entrance of the dye penetrant into the damage zone after impact analysis. The post-repair compression strength after impact testing showed about a 10% strength improvement [102,104].

It is challenging to incorporate self-healing characteristics into a material without meaningfully altering its physical and mechanical characteristics. For example, an addition of fibres, microcapsules, or micro-vascular networks may modify the elasticity or toughness of the prime material significantly. Because of this, one may favour an intrinsically repairing material over an extrinsically repairing material. However, there are other drawbacks of using intrinsically healing materials. As an illustration, other dynamic bond-based self-healing mechanisms can only be realistic for a specific polymer, whether it is a polymer with a low *T*_g_, a thermoset, a polymer at basic pH values, or an ionic polymer. Another disadvantage of materials depending on dynamic bonds, either covalent or non-covalent bonds, is that damage on the nanoscale must be distributed only on the dynamic bonds in order to allow 100% repair and recovery of mechanical properties. However, repair using dynamic bonds can only accommodate so much of the damage that is sustained by non-dynamic bonds before the mechanical strength of the structure weakens [74].

## 5. Fracture Mechanics of Polymeric Materials

### Structural Aspect of Composite Failure

There are several reinforcements in composites that consist of brittle fibres (i.e., glass or carbon) [105,106], in a brittle polymer matrix, such as epoxy or polyester resin. Because of their heterogeneous nature and construction, composite materials have improved mechanical properties [105], but propagation of a crack is more complex than that in homogeneous materials. 

The fracturing of a composite involves the breaking of the load-bearing fibres and the weak matrix, and a complex mixture of crack deviances along these weak interfaces [107,108]. The fracture of these materials is not a simple process because of the microstructural heterogeneity and anisotropy of fibre composites. Although the complex combination of micro failure leads to a weakening of the load-bearing ability, it is also responsible for high levels of toughness, and the same complexity makes it problematic to use techniques based on fracture mechanics for design purposes [107]. The compiled geometry of a composite influences crack spreading, because some laminates seem to be extremely notch-sensitive while others are absolutely unsusceptible to the presence of stress concentrators. The choice of resins and fibres, the manner of combination in the composite, and the quality of the industrially made composite should all be judiciously organised if ideal toughness is to be achieved [105,106,107,108,109]. Moreover, materials conditions for the optimum tensile and shear strengths of the laminates are rarely compatible with the prerequisites for the highest toughness. Research into the fracture mechanics of composites is in its early stages, in comparison to fracture mechanics in homogeneous material such as metals. The basic research background has not yet been completely established, and there is no simple formulation for computing the toughness of all composites. Engineers and scientists are unable to design with confidence the structure of any composite so as to deliver the optimal arrangement of strength and toughness [106,110].

In metallic and plastic structures, even moderately fragile ones, energy is scattered in non-elastic distortion mechanisms in the section of the crack tip. This energy is lost in moving displacements in metallic and viscoelastic flow development in a polymer. In composites, the fibres affect crack progression, but their consequence depends on the strength of the bond between the fibre/matrix (resin). For instance, if the fibre/matrix bond is robust, crack may propagate via both the fibre and the matrix with consistency. In this case the composite toughness would be low and roughly equivalent to the quantity of the distinct module toughness. On the contrary, if the fibre/matrix bond is weak, crack transmission becomes difficult and other deformation mechanisms may influence to the complete fracture mechanism of the composite [107].

## 6. Early Facture Testing

### 6.1. Linear Elastic Fracture Mechanics

Fracture mechanics is a failure theory that uses energy principles, possibly in conjunction with strength criteria, and takes into account failure propagation through the structure [111]. The primary theory of fracture called linear elastic fracture mechanics (LEFM) is an uncompromised body of knowledge. LEFM is the basic theory of fracture, initially established by Griffith (1921–1924) and finalized in its critical form by Irwin and Rice [110]. LEFM is a straightforward, although complex, concept that deals with sharp cracks in elastic forms [112]. The basic fundamentals of fracture mechanics may be summarized as manner of a triangle with three critical constraints positioned at each apex: operational stress, fracture toughness and critical defect size [113].

LEFM presents an exceptional quantifiable depiction of both a crack’s mobility and of the elastic fields enclosing its tip [114,115]. Here, assumptions underlying the validity LEFM are only the dynamic behaviour of straight distinct crack states are accounted for, but LEFM cannot correctly calculate the behaviours of complex dynamic states like micro-branching. Presently, there is no first method guidance for what route a crack needs to take [115]. In LEFM, it is assumed that linear elasticity is viable away from the crack tip. Fundamentally, for enormous samples, this hypothesis can constantly be made as precise as desired, but for fixed sized samples it is essential to check the linear elasticity [115,116]. Moderate production is assumed, all nonlinear and/or dissipative procedures are presumed to occur in a section of insignificant size at the tip of a crack. LEFM is a scale-free model, thus, the measures at which these multifaceted procedures occur are insignificant [117]. The actuality of added scales inside the crack tip area has a great effect on crack dynamics. The general assumption regarding the energy balance is that energy has to move into a crack tip, or else a crack cannot spread [115].

#### 6.1.1. Griffith Theory of Brittle Fracture

An examination of the initial fracture was established using the energy method that defines the occurrence of fracture when the energy accessible for crack evolution is adequate to overcome the resistance of the material. Griffith was the first to recommend the essential energy principle for inelastic materials like glass, where the resistance was presumed to arise completely from the surface development energy of the structure [105,106,118]. It is imperative to recognise that the Griffith relation was derived for an ideal elastic material containing a very sharp crack.

Griffith’s concept states that solids possess surface energy, thus, for the propagation of a crack, the parallel surface energy must be balanced through the externally supplemented or internally discharged energy. For a linear elastic solid, this donated energy, which is essential for increasing the crack, might be estimated from the resolution of the consequent crack dilemma. Using Inglis’ explanation for a homogeneously loaded plate with an oval void, Griffith premeditated the growth in strain energy and, from the energy balance, acquired the stress equivalent to fracture as [119,120]:

(1) $$\sigma ={\left(\frac{2\gamma_{s}E^{*}}{\Pi a}\right)}^{1/2}$$

where $E^{*}$ = E for a plane stress and $E^{*}$ = $\frac{E}{\left(1-v^{2}\right)}$ for the plane strain condition, $\gamma_{s}$ is the specific surface energy, a is the half crack length, E is the elastic modulus, σ is the applied stress and v is the Poisson’s ratio. Griffith was able to interpret the infinite stress inconsistency acknowledged by Wieghardt and to demonstrate that the fracture stress is reliant on the flaw size through the equation $\sigma =\frac{m}{\surd a}$, where m is the material constant [119]. 

#### 6.1.2. Irwin and Orowan’s Modification to Griffith Theory of Brittle Fracture

The Griffith theory is strongly dependent on the magnitude of the crack and satisfies only ideally brittle materials like glass. Thus, Irwin and Orowan explained that in common engineering materials the energy used in creating new surfaces had two constituents: initially, the typical surface energy expression studied by Griffith, and secondly, the energy absorbed by plastic distortion, the latter being dominant.

Therefore, Irwin and Orowan independently modified Griffith’s theory to take into account the plasticity constant which was observed to accompany fracture in metals. Equation (1) was revised with an additional term, $\gamma_{p}$, the plastic work per unit area of the new surface created:

(2) $$\sigma ={\left(\frac{2E^{*}\left(\gamma_{s}+\gamma_{p}\right)}{\Pi a}\right)}^{1/2}$$

However, $\gamma_{p}$ is typically greater than $\gamma_{s}$, and it relates to the energy dissipated by dislocation movement within the material near the crack. Therefore, the modified Griffith’s equation can be written as follows:

(3) $$\sigma ={\left(\frac{2E^{*}\left(\gamma_{s}+\gamma_{p}\right)}{\Pi a}\right)}^{1/2}\approx \;(\frac{E^{*}2\gamma_{p}}{\Pi a}{)}^{1/2},\;\mathrm{when}\;\gamma_{p}>>\gamma_{s}$$

Irwin’s second modification to the Griffith theory was replacing the term $2\gamma_{p}$ with the potential strain energy release rate G. He showed that G is measurable and can relate to the stress intensity factor, K, obtained from the examination of the sharp crack fracture toughness. The critical condition at which the crack spreads to cause a global failure is when the value of G exceeds the critical value.

(4) $$\sigma ={\left(\frac{GE^{*}}{\Pi a}\right)}^{1/2}$$

Sneddon’s study on a plane and axisymmetric crack used Westergaard’s resolution for plane problems and by resolving a penny-shaped crack. Sneddon acquired the precise asymptotic behaviour of the stress field close to the crack tip and presented that the outcome of the two circumstances varies only by an arithmetic factor of $\frac{2}{\Pi }$ [119]. For the penny-shaped crack, Sneddon also got a correct expression of Griffith’s energy balance equation [120,121,122]. However, Sneddon inaccurately declared that $\sigma_{\theta \theta }$, which seems in the axisymmetric problem has no analog in the plane problem, therefore, Sneddon failed to recognize the universal nature of the crack tip stress field. Irwin later indicated, asymptotically, that the stress formed around the boundary of the penny-shaped crack is one of plane strain and the recognition of the ‘numerical factor’ perceived by Sneddon it is a crucial aspect in simplifying the results. Irwin’s key influence, therefore, was an identification of the universal nature of the asymptotic stress and displacement fields around the crack interior in linear elastic solid. It was reported that the symmetric crack clarifications specified by Sneddon and Westergaard might be simplified to incorporate the asymptotic quantified for all crack problems and, for a small distance from the crack tip [119], the stresses might be defined as:

(5) $$\sigma_{ij}≅\left(\frac{K}{\sqrt{2\Pi r}}\right)f_{ij}\left(\theta \right)$$

where $f_{ij}$ is the function obtained earlier by Wieghardt, Westergaard and Sneddon for a specific crack geometries and loading condition and K is the stress intensity factor.

#### 6.1.3. Stress Intensity Factor

The stress intensity factor [123] concept was initially established in the framework of fracture mechanics. For fracture considerations, both brittle fracture and fatigue failure, the asymptotic singular stress field at the directed crack is critical. The stress level around the singularity is described by the stress intensity factor (SIF), perhaps overlaid by the crack parallel to the nonsingular T-stress. In particular, in circumstances such as thin-sheet lap joints, it is essential, to take the complex order expressing of the stress field estimate into account [124].

**Figure 10:**
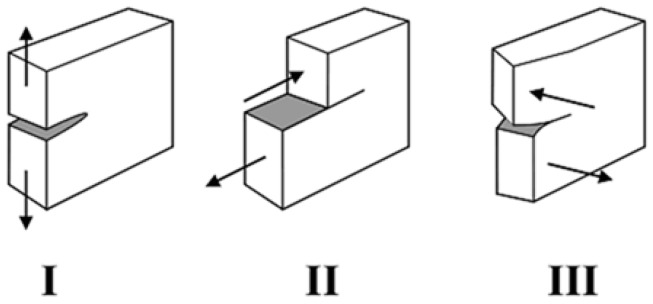
Characterization of three distinct fracture methods: mode I is an in-plane opening; mode II is an in-plane shear; and mode III is an out-of-plane shear loading.

The local three-dimensional stress originality at a specific point of the crack or slit front is usually defined by the superimposition of three two-dimensional stress individualities analogous to three unrelated loading or opening methods of the crack tip (see Figure 10): mode I is in-plane opening, mode II is in-plane shear and mode III is the out-of-plane shear loading [125]. The related SIFs are KI, KII and KIII [124]. Non-singular stresses might be overlaid, and these stresses include the crack-parallel stresses (the T-stress), normal stresses and the symmetric shear stresses in the crack front direction. In usual instances, separation of the rudimentary slit tip loading modes is impossible, linking effects with the transverse singular effects occur, for example, where the crack front butts on a free surface [124,125].

Three types of fractures define the relative motion through a fracture close to its boundary: modes I, II, and III (i.e., Figure 10). In mode I, which is referred to as the in-plane opening, the frame is loaded by tensile forces, so that the crack surfaces are torn separately in the *y*-direction. The distortions are then equal with regard to the planes perpendicular to the *y*-axis and the *z*-axis [126,127,128]. In the in-plane shear mode or mode II, the body is loaded by shear forces parallel to the crack surfaces, which glide over each other in the *x*-direction. The distortions are then symmetric with respect to the plane perpendicular to the *z*-axis and skew equal with regard to the plane perpendicular to the *y*-axis [129]. Lastly, is the out-of-plane shear loading which is also known as mode III, the body is loaded by shear forces parallel to the crack front of the crack surfaces, and the crack surfaces glide over each other in the *z*-direction. The deformations are then skew-symmetric with regard to the plane perpendicular to the z and the *y*-axis [115,128,129].

## 7. Modelling

Theoretical modelling and application of computational design devices to calculate the attributes of self-healing materials are still in their infancy. The modelling attempts to relate self-healing of thermoplastics and numerous facets of thermoset materials have been discussed, but with a strong focus on the microencapsulation methodology in recent journals [31]. The concept of modelling self-healing materials was first described in the early 1980s, which focus on understanding the processes in damage and the repair of thermoplastics [14]. Five phases of crack repair in thermoplastics were represented as: (i) the surface reorganization stage, which starts the diffusion function; (ii) the method phase directs the manner of restoration; (iii) the wetting step shapes the wetting diffusion function; (iv) the diffusion phase is thought to be as the most significant stage where restoration of mechanical properties occurs; and (v) the randomization phase includes a complete loss of memory of the crack interface [14,31]. While this model is suitable for thermoplastics, the opposite was observed for thermoset materials because the chain mobility of the former is expected to fit into the five phase repairing classification [31].

In recent years the emphasis has shifted to the development of self-healing materials of thermoset-based structures [130,131], in conjunction with the latest modelling trends to model characteristics of these self-healing materials. Three modelling concepts for thermoset based self-healing have been studied; these include micro-mechanical modelling, continuum damage mechanics, and, cohesive modelling.

White et al. reported on the effect of microcapsule geometry and properties on the mechanical activating procedure. Design parameters such as the toughness, the comparative stiffness of the microcapsules, and the strength of the interface amongst the microcapsule and the matrix were investigated. Micromechanical modelling with the aid of the Eshelby–Mura equivalent inclusion technique was applied to study numerous characteristics of the compound three-dimensional correlation between a crack and a microcapsule [21,31]. Optical and scanning electron microscopy was used to established the repair theory [21]. The second modelling concept for self-healing material is the continuum damage mechanics. This concept of modelling was pioneered by Kachanov in 1958 [132,133]. This was extended to simulate the irreversible repairing mechanism, which accelerated the advancements of a numerical model of damage, plasticity, and repair for fibre-reinforced polymer composites [31]. The third modelling concept is cohesive modelling, which is recognized as a method that is used to simulate different crack growths in a unified system under complicated geometric and loading conditions [134]. The cohesive modelling of fracture assumes that fracture development occurs in a vanishingly thin region ahead of the crack tip. This method seems suitable for summarising the failure of polymeric structures, where the growth of a thin crazing zone is an important constituent. Cohesive models have been researched for quasi-static and dynamic crack transmission in polymers [135]. Yang et al. defined a mixed mode cohesive zone model (CZM), which led to correct predictions of mixed mode fracture (i.e., mode I, mode II, and mode III) of adhesively bonded joints with large non-linear deformation. The CZM uses autonomous cohesive laws for the opening mode (mode I) and shear modes (modes II and III), such that the durability and cohesive strengths for the three modes can be diverse [108]. Maiti et al. used molecular-scale level simulation to investigate the healing kinetics of a healing agent, to correlate precisely the relation between the mechanical reaction of the repairing agent and its structure as a function of the degree of treatment. In their approach three levels are incorporated. First, is a fully atomistic level, where all atoms restricted in the system are absolutely characterised at this level where reaction rates and structural information can be defined, the second phase, is the first coarse-graining level, where larger polymeric systems are simulated and the connection between structure and localized mechanical attributes are recognised, and last, is the second coarse-graining level, in which reaction rates and damages degrees are resolved and an inclusive mechanical response of the repairing agent under the process circumstances is established [136].

However, advancement in this area can eventually enable the manufacture of robust materials that can both screen their structural reliability and heal themselves before any disastrous failure occur. What is still needed in the scientific community are operative principles to improve the design and allows the effective synthesis of these materials. It is in this area that theoretical and computational modelling can play an important role. Particularly, simulations models for fluid interactions can enlighten researchers about the dynamic behaviour at the interface of a fluid-packed network and the surrounding solid structure. Furthermore, models can disclose how to plan receptive polymeric materials that vigorously contribute in reinforcing the arrangement when it is damage or subjected to different environmental conditions [137]. While these models have vastly contributed to our understanding of crack propagation in composite materials, they have drawbacks when used alone. For example the continuum modelling technique does not quantify morphology and transport characteristics of the healing agent, whereas, the cohesive finite element technique hypothesizes immediate repairing, compared to the experimental adherence of rest periods on the fatigue response that achieved repair [31]. A good model should address the fracture of composite laminates, it is prompted by the beginning and development of four failure modes such as matrix cracks, fibre breakage, delamination between neighbouring layers of the laminate and interfacial debonding [138,139]. There are four stages of deformation in composites. The first stage is where the matrix debonds from the fibre ends, regularly at an initial point of loading. It does not automatically advance to the other damage instantaneously, but rather triggers localized plastic deformation. In the second phase the matrix cracking and fibre/matrix interface debonding are caused by the stress concentration from the debonded fibre end. The boundary crack and/or matrix crack transmits with amplified loading. In the third phase the individually fashioned micro-cracks cleave and form macroscopic cracks. The fourth phase is where a macroscopic crack spreads unsteadily, causing final failure. Materializing these descriptive interpretations into measurable micro-mechanics models is difficult [139].

Illustrations where computational theories have provided strategies for manipulating self-healing structures were highlighted above, and suggest new areas for further examination. While there have been several attempts in this area, theoretical and computational research into self-healing in synthetic materials is still in its infancy. Advancement in this area requires an improvement of effective models that can explore the structural progression of multicomponent, multiphase structures. At the same time, the models should not only describe how the mechanical forces in the system relate to the structure, but also consequential structural alterations modify the mechanics [140].

The first fundamental problem with advanced materials and composites used for engineering structures, spacecraft, and vehicle applications is that they are rigid solids. It is into these materials that the self-healing characteristics must eventually be used to improve the durability and safety of these materials. Therefore, the essential design and fabrication challenge is to introduce fluidity into these systems and do so without changing the mechanical attributes of the structure. A solution proposed was to implant a three-dimensional vascular network inside the structural materials. Such research has recently appeared in some publications [141,142], where vascular fibres channels spread throughout the structures and contain repairing agents. When a crack occurs, the healant flows to the deformed area and seal the crack. Such integrated system present opportunities in the establishment of these autonomous and continuous healing materials. However, operative standards to improve the design and allow effective synthesis of these materials are still needed. Particularly, computational representations for fluid-structure relations may offer information about the dynamic behaviour at the interface of a fluid-packed network and the surrounding solid walls. Furthermore, models can disclose how to design responsive polymeric materials that vigorously contribute in reinforcing the structure when it is damaged or exposed to differences in environmental situations.

## 8. Conclusions

This study has sought to provide information on studies conducted on self-healing polymeric and polymeric composites materials. Polymeric and polymeric composite materials are widely used globally because of their cost effectiveness and availability. Methods of integrating self-healing competencies in polymeric structures can now efficiently tackle several deformation mechanisms at molecular and structural phases. Although highly significant information has been reported for self-healing properties in different materials, limited information is available on self-healing polymeric/composites. The inherent complications of their damage restoration are still limiting their use in various applications. In particular, if repeated healing is incorporated in these polymer/composite materials, it would make them smart materials with multi-functional capabilities (i.e., self-diagnosis, self-control and self-healing). Effective strategies are highly desired for improving the design, and facilitating the proficient synthesis and application, of polymeric/composite systems. It is in this regard that theoretical and computational modelling shows a promising solution, particularly, computational simulations for fluid-flow relations, which can provide information about the dynamic behaviour at the interface of a fluid packed network and the surrounding solid structure. However, before engineers can focus on computational modelling, they need to comprehend and consider the fundamental nature of fracture (i.e., fracture formation and propagation); these fundamentals are significant research in the material scientific society which will present essential theories for modelling self-healing polymer/composites. Although significant advancement has been made in the past several years, the field is still in its infancy. Many of the ambiguous fundamental concerns like environmental degradation, oxidation and elevated temperature behaviour are multifaceted and need an understanding and analysis of the topic from different perspectives such as material science, electrochemistry and solid mechanics.
